# Examination of postural dependence in subcutaneous fat thickness measurement using a caliper

**DOI:** 10.1186/s40101-026-00435-9

**Published:** 2026-06-15

**Authors:** Tomoyuki Yamauchi, Atsushi Sugama

**Affiliations:** Department of Science and Technology, Seikei University, 3-3-1 Kichijoji Kitamachi, Musashino-Shi, Tokyo, 180-8633 Japan

**Keywords:** Metal caliper, Body position, Lumbar region, Lower leg

## Abstract

**Background:**

Standing is commonly used as the standard position for skinfold-based subcutaneous fat thickness measurement; however, the feasibility and reliability of alternative positions remain unclear.

**Methods:**

Ten healthy men participated. Subcutaneous fat thickness was measured at the lumbar and medial calf regions using a metal caliper. Measurements were obtained in standing, sitting, prone, side-lying, and supine positions, except for the lumbar region. Each posture was measured three times in one session by the same examiner, and participant-level means were used for analysis. Intra-rater reliability was assessed using ICC(1, 1) and ICC(1, 3).

**Results:**

Participant-level means ranged from 3.7 to 4.0 mm in the lumbar region and from 4.2 to 4.4 mm in the lower leg. ICCs exceeded 0.90 in both regions. A main effect of posture was observed in the lumbar region, whereas the Greenhouse–Geisser-corrected main effect was not significant in the lower leg. Post hoc differences were found only between standing and side-lying and between standing and prone positions in the lumbar region, with Cohen’s d values of 0.19 and 0.16, respectively.

**Conclusions:**

Subcutaneous fat thickness measurements obtained using a metal caliper showed very high intra-rater reliability across postures. Although small posture-related differences were observed, particularly in the lumbar region, the magnitude of these differences appeared to be of limited practical relevance in healthy young men. Alternative measurement postures may be considered when standing measurement is difficult, although further validation in other populations is required.

## Background

Low back pain is one of the most common work-related musculoskeletal disorders, and trunk muscle fatigue during sitting has been widely studied [1]. Muscle fatigue is often assessed using the median frequency obtained from surface electromyography (neural fatigue) [1, 2]. In addition, muscle oxygen saturation and total hemoglobin concentration measured by near-infrared spectroscopy have been reported to be associated with muscle fatigue and tissue blood flow [3, 4] and have been used to analyze metabolic or hemodynamic responses in lumbar and lower-limb regions [5–7].

In spatially resolved spectroscopy, a form of near-infrared spectroscopy, near-infrared light is emitted into biological tissues, and the reflected light intensities at different source-detector distances are measured. The slope of the attenuation of these light intensities provides information about the muscle tissue. The optical properties of the skin at the measurement site have little effect on spatially resolved spectroscopy measurements. However, when the subcutaneous fat layer is thick, the penetration depth of light into the muscle tissue decreases, and most of the reflected light originates from adipose tissue. Therefore, correction using measured subcutaneous fat thickness is required [8]. Subcutaneous fat thickness is commonly assessed using ultrasonography or the skinfold method with a caliper. Previous studies have reported that values obtained using ultrasonography, metal calipers, and magnetic resonance imaging may not differ substantially at certain measurement sites [9, 10]. However, inter-device correlation does not necessarily indicate agreement. In particular, although metal caliper measurements can be highly correlated with ultrasonographic measurements, insufficient agreement and systematic overestimation by metal calipers have also been reported [11]. In addition, the durability and repeated use of calipers are relevant methodological considerations; plastic calipers may deform with long-term use, whereas metal calipers are recommended for research purposes because of their greater durability [12]. Thus, measurement site, inter-device agreement, and device durability should be considered separately when selecting a method for assessing subcutaneous fat thickness.

Most standardized anthropometric protocols, including those of the International Society for the Advancement of Kinanthropometry, are based on standing positions [13]. Subcutaneous fat thickness can be measured at various anatomical sites, such as the upper arm, abdomen, and medial calf. For example, for the medial calf site, the participant typically stands with the measured leg placed on a platform and the hip and knee joints flexed. However, this posture may not be optimal for all examiners or participants. In clinical and physiological research settings, some individuals may have difficulty maintaining a stable standing posture because of pain, frailty, neurological impairment, balance problems, or lower-limb dysfunction. In such cases, measurements may be attempted in more stable positions, such as sitting or supine, but generally accepted alternative postures or correction methods for metal caliper measurements have not been established. Clarifying whether posture affects metal caliper-derived subcutaneous fat thickness would therefore help expand the applicability of the measurement in both clinical practice and physiological research.

To date, only one study has examined the effects of body position on measurement reliability and values using ultrasonography [14]. However, although a metal caliper offers a simple and cost-effective alternative to ultrasonography for measuring subcutaneous fat thickness, the influence of body position on measured values and reliability remains unclear. Therefore, this study aimed to examine the effects of different body positions on measured values and reliability of subcutaneous fat thickness assessments using a metal caliper. We hypothesized that the reliability of subcutaneous fat thickness measurements obtained using a metal caliper would be high across different body positions and that the differences in the measured values between these positions would be small.

## Methods

### Aim, design, and setting of the study

A cross-sectional reliability and comparison study was conducted to examine posture-related differences in subcutaneous fat thickness measured using a metal caliper. All measurements were performed in an indoor university laboratory under air-conditioned room-temperature conditions.

### Participants

Ten healthy adult male volunteers (age: 21.7 ± 0.8 years, height: 173.3 ± 5.0 cm, weight: 62.5 ± 13.3 kg, body mass index [BMI]: 20.7 ± 4.0 kg·m^2^) participated in this study. The inclusion criteria were the absence of exercise and alcohol consumption within 12 h prior to the measurement. The exclusion criteria were the presence of pain during movement; a history of low back or lower limb pain within the past 6 months; spinal disorders; neurological deficits, such as muscle weakness, sensory loss, or sensory changes; osteoarthritis; rheumatoid arthritis; gout; renal disease; open wounds or contusions on the lower back or lower limbs; and any low back pain or sprains of the knee or ankle joints requiring medical treatment. All participants underwent subcutaneous fat thickness measurement on the same day.

This study was conducted in accordance with the principles of the Declaration of Helsinki and adheres to the STROBE guidelines for observational studies. This study was approved by the Ethics Committee of Seikei University (SREC 25–13). Written informed consent was obtained from all participants before enrolment.

### Measurement sites

The measurement sites were the lumbar region and the medial calf region of the lower leg. The terminology and anatomical definitions were standardized to align with ISAK terminology where applicable. The medial calf site was defined as the point of maximal calf circumference on the medial aspect of the right lower leg, corresponding to the medial gastrocnemius belly [15]. Because the lumbar region is not a standard ISAK skinfold site, it was defined as a study-specific site over the right multifidus muscle. The multifidus muscle was identified at the intersection of the line connecting the posterior superior iliac spine and the spinous process of the first and second lumbar vertebrae, corresponding to the level of the fifth lumbar spinous process [16].

### Measurement postures

**Fig. 1:**
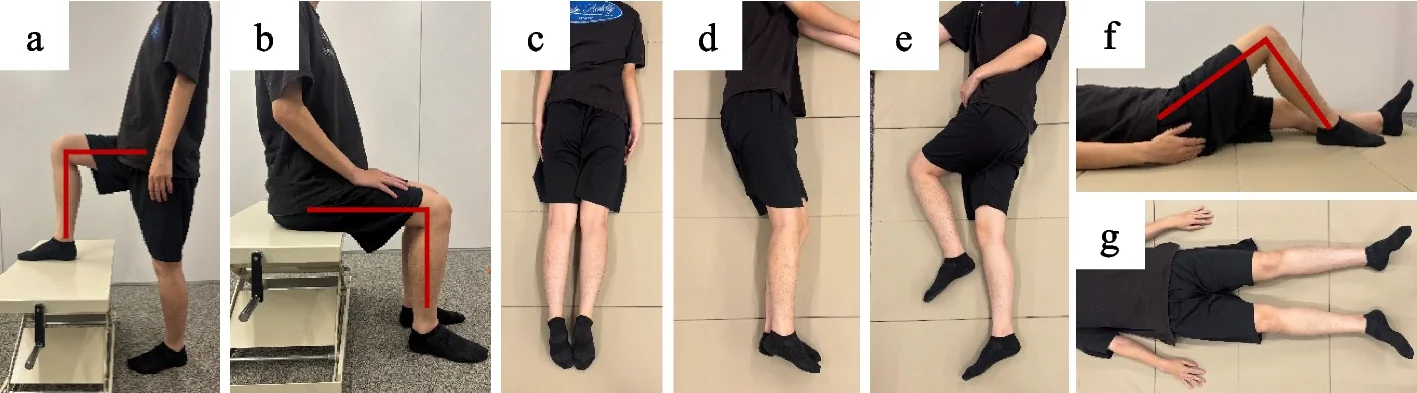
Measurement postures. **a** Standing posture. **b** Sitting posture. **c** Prone posture. **d** Side-lying posture (lumbar). **e** Side-lying posture (lower leg). **f** Supine posture (knee flexion). **g** Supine posture (knee extension)

For the lumbar region, measurements were performed in the standing, sitting, prone, and side-lying positions. For the lower leg, measurements were conducted in the same postures as for the lumbar region, with the addition of two supine positions: knee flexion and knee extension. In the standing position, the participants stood naturally for lumbar measurements. For lower leg measurements, the participants stood on a height-adjustable platform with the right hip and knee flexed at 90° while keeping the trunk and left lower limb vertical. In the sitting position, the participants sat on a height-adjustable platform with both hips and knees flexed at 90°, trunk upright, and soles of the feet in full contact with the floor. In the prone position, the ankle joints were maintained in a relaxed plantar-flexed position. In the side-lying position, for lumbar measurements, the participants lay on their left side with the right side facing upward. The lower limbs were aligned with the trunk, and both shoulders were flexed at approximately 60° to maintain the posture. For lower leg measurements, the participants lay on their left side with the right leg positioned downward. The right lower limb was aligned with the trunk, the left hip was flexed at 45°, and the left lower leg was parallel to the right one and in contact with the floor. In the supine knee-flexion position, the right knee was flexed at 90°, with the right sole in full contact with the floor. In the supine knee-extension position, both lower limbs were naturally extended with the feet placed at pelvis width, and both ankle joints were relaxed (Fig. 1).

### Subcutaneous fat thickness measurement

Subcutaneous fat thickness was measured using a calibrated metal skinfold caliper (skinfold caliper, T.K.K. 5011a, SANKA Co., Japan; measurement range: 0–60 mm; resolution: 1 mm; constant pressure: 10 g/mm^2^) according to the skinfold method. The surfaces of the caliper were cleaned with alcohol tissues before use, consistent with anthropometry equipment care procedures [17]. If the participant's skin was visibly moist, the measurement site was gently wiped with an alcohol tissue and allowed to dry before measurement. At the marked measurement site, the skin and subcutaneous tissue were grasped between the thumb and index finger, and the fold was gently moved laterally to confirm the absence of muscle tissue. The nearest edge of the caliper jaws was positioned 1 cm from the fingers at the midpoint of the pinched skinfold. The caliper was always held perpendicular to the skinfold surface. The measured value was recorded 2 s after the full pressure of the caliper was applied [18, 19]. The smallest readable unit on the scale was 0.5 mm. If the indicator needle was positioned midway between two scale marks, the value was recorded as 0.5 mm; if the needle clearly leaned toward one mark, the integer value of that mark was recorded. The recorded value was divided by two to obtain the subcutaneous fat thickness [10]. All measurements were performed by the same examiner (a licensed physical therapist). All measurements were conducted on the same day within a single session. For each posture and measurement site, three nonconsecutive measurements were obtained, and the arithmetic mean of the three measurements was used as the representative value for each participant.

### Statistical analysis

Statistical analyses were performed using SPSS version 29 (IBM Corp., Armonk, NY, USA), with a significance level of 5%. The normality of all variables was assessed using the Shapiro–Wilk test. The intra-rater reliability of the measurements was evaluated using intraclass correlation coefficients, ICC (1, 1) and ICC (1, 3). The three repeated measurements obtained for each posture and measurement site were used for the ICC analysis. ICC (1, 1) was calculated to estimate the reliability of a single measurement, whereas ICC (1, 3) was calculated to estimate the reliability of the mean of three repeated measurements. For subcutaneous fat thickness, differences among postures were analyzed using one-way repeated-measures analysis of variance (ANOVA); the arithmetic mean of the three repeated measurements was used as the representative value for each participant. Mauchly's test was used to assess sphericity; when sphericity was violated, Greenhouse–Geisser-corrected degrees of freedom and *p*-values were used. When a significant main effect was found after any required correction, post hoc pairwise comparisons were performed with Bonferroni correction for multiple comparisons. *p*-values were reported to three decimal places, except when *p* < 0.001.

The number of participants was determined based on the sample size calculation required for statistical analysis. As no previous studies have reported the effect size for the lumbar region, the assumed effect size was set to Cohen's f = 0.20. For the lower leg, the effect size calculated from data reported in the previous ultrasonography posture study was extremely large (f = 7.56) [11]; therefore, this value was not used directly for power analysis. Accordingly, a large effect size of f = 0.40 was assumed for the sample size calculation. The sample size was estimated using G*Power for repeated-measures ANOVA (within factors). For the lumbar region, the parameters were set as follows: effect size f = 0.20, α error probability = 0.05, prespecified statistical power = 0.80, number of groups = 1, number of measurements = 4, and correlation among repeated measures = 0.90, which was adopted based on the previous ultrasonography posture study in the absence of prior reports for the lumbar region [11]. Under these conditions and assuming sphericity, the required sample size was nine participants, and the achieved power was 0.84. For the lower leg, the parameters were as follows: effect size f = 0.40, number of measurements = 6, correlation among repeated measures = 0.57 [11], and an assumption of sphericity. The required sample size was seven participants with a prespecified statistical power of 0.80. Considering possible experimental variability and participant dropout, 10 participants were included to ensure adequate statistical power and stability of the results.

## Results

The ICCs for subcutaneous fat thickness measurements in each posture are presented in Table 1. The three repeated measurements obtained for each posture and measurement site were used for the ICC analysis. ICC (1, 1), which reflects the reliability of a single measurement, and ICC (1, 3), which reflects the reliability of the mean of three repeated measurements, were both very high. The ICC (1, 1) values for the lumbar region ranged from 0.981 to 0.990 (95% CI: 0.948–0.997), and the ICC (1, 3) values ranged from 0.994 to 0.997 (95% CI: 0.982–0.999). The ICC (1, 1) values for the lower leg ranged from 0.981 to 0.995 (95% CI: 0.948–0.999), and the ICC (1, 3) values ranged from 0.994 to 0.998 (95% CI: 0.982–1.000).

**Table 1:** Intra-rater reliability of subcutaneous fat thickness measurements in the lumbar and lower leg regions in each posture

Measurement posture	Intraclass correlation coefficient (ICC)	95% confidence interval (95% CI)
Lumbar region		
Standing posture	(1, 1) 0.981	0.948—0.995
	(1, 3) 0.994	0.982—0.998
Sitting posture	(1, 1) 0.987	0.964—0.996
	(1, 3) 0.996	0.988—0.999
Prone posture	(1, 1) 0.988	0.967—0.997
	(1, 3) 0.996	0.989—0.999
Side-lying posture	(1, 1) 0.990	0.973—0.997
	(1, 3) 0.997	0.991—0.999
Lower leg region		
Standing posture	(1, 1) 0.983	0.954—0.995
	(1, 3) 0.994	0.984—0.998
Sitting posture	(1, 1) 0.992	0.978—0.998
	(1, 3) 0.997	0.993—0.999
Prone posture	(1, 1) 0.995	0.986—0.999
	(1, 3) 0.998	0.995—1.000
Side-lying posture	(1, 1) 0.995	0.985—0.999
	(1, 3) 0.998	0.995—1.000
Supine posture (knee flexion)	(1, 1) 0.993	0.979—0.998
	(1, 3) 0.998	0.993—0.999
Supine posture (knee extension)	(1, 1) 0.994	0.984—0.998
	(1, 3) 0.998	0.995—0.999

The mean subcutaneous fat thickness values for each posture are listed in Table 2. These values represent the arithmetic mean of three repeated measurements for each participant, summarized across all participants. The mean values for the lumbar region ranged from 3.7 to 4.0 mm, and those for the lower leg ranged from 4.2 to 4.4 mm.

**Table 2:** Subcutaneous fat thickness values in each posture (participant-level mean of three repeated measurements, mean ± SD)

Measurement posture	Lumbar	Lower leg
Standing posture (mm)	4.0 ± 1.1	4.2 ± 2.1
Sitting posture (mm)	3.9 ± 1.0	4.2 ± 2.0
Prone posture (mm)	3.8 ± 1.0	4.4 ± 2.2
Side-lying posture (mm)	3.7 ± 1.0	4.4 ± 2.2
Supine posture (knee flexion) (mm)	-	4.2 ± 2.0
Supine posture (knee extension) (mm)	-	4.3 ± 2.2

Values represent the mean of three repeated measurements calculated for each participant and then summarized across participants

**Fig. 2:**
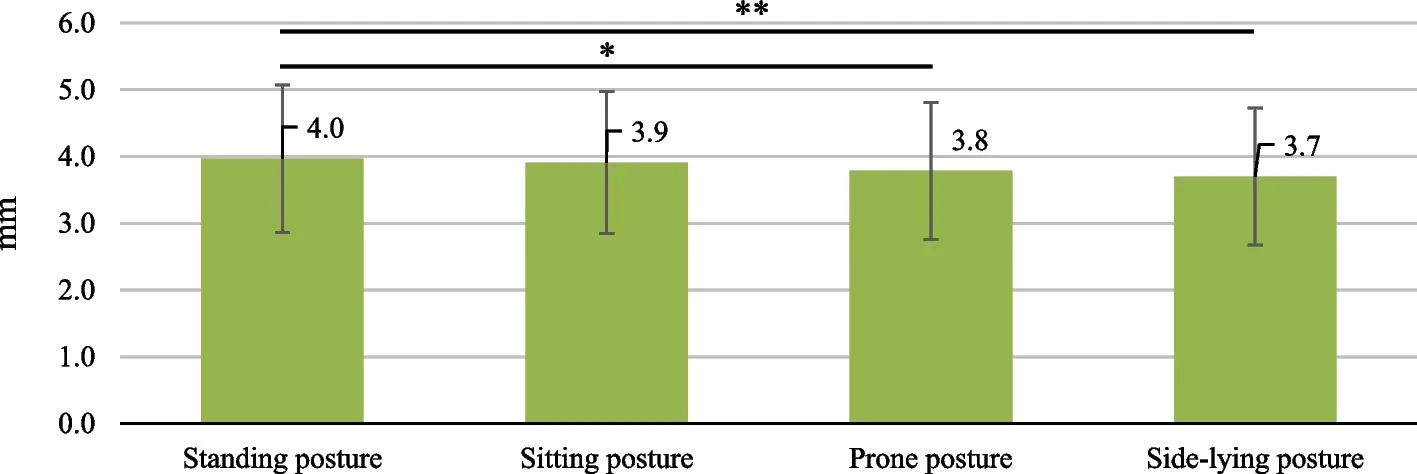
Multiple comparison tests for subcutaneous fat thickness of the lumbar region among postures. Values are shown as mean ± SD. *, *p* < 0.05; **, *p* < 0.01

**Fig. 3:**
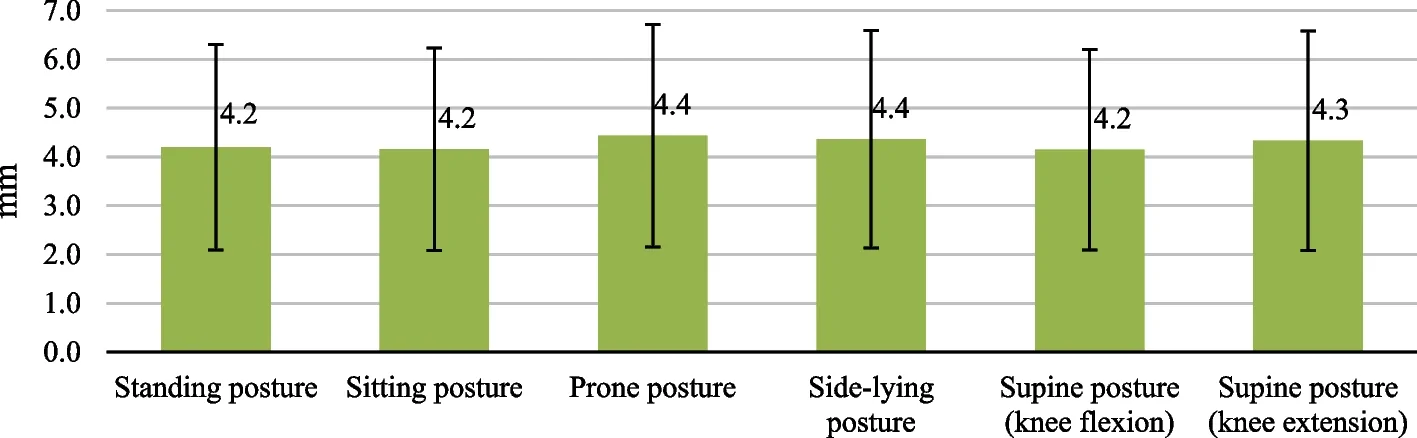
Multiple comparison tests for subcutaneous fat thickness of the lower leg region among postures. Values are shown as mean ± SD

The results of the one-way repeated-measures ANOVA are presented in Table 3. For the lumbar region, Mauchly's test did not indicate a violation of sphericity (W = 0.514, *p* = 0.402), and a significant main effect of posture was observed (F (3, 27) = 5.51, *p* = 0.004, partial eta^2^ = 0.38). Post hoc pairwise comparisons revealed significant differences between the standing and side-lying positions and between the standing and prone positions (*p* = 0.008 and *p* = 0.030, respectively). Subcutaneous fat thickness in the standing position was significantly greater than in the side-lying position (Cohen’s d = 0.19) and prone position (Cohen’s d = 0.16) (Table 4, Fig. 2). Across all lumbar pairwise comparisons, Cohen’s d ranged from 0.03 to 0.19. For the lower leg, Mauchly's test indicated a violation of sphericity (W < 0.001, *p* < 0.001); therefore, Greenhouse–Geisser correction was applied. The corrected main effect of posture was not significant (F (1.27, 11.46) = 2.47, *p* = 0.139, partial eta^2^ = 0.22). Pairwise comparison results for the lower leg are shown descriptively in Table 4, and no significant differences were found among postures (all *p* > 0.100; Cohen's d = 0.00–0.10) (Fig. 3).

**Table 3:** One-way repeated-measures ANOVA for measurements in the lumbar and lower leg regions in each posture. Greenhouse–Geisser (GG) correction was applied to the lower-leg analysis because Mauchly's test indicated violation of sphericity

Factor	Degrees of freedom (df)	F-value	*p*-value	partial η^2^
Lumbar region				
Position	3, 27	5.51	0.004	0.38
Lower leg region				
Position (GG corrected)	1.27, 11.46	2.47	0.139	0.22

**Table 4:** Bonferroni-adjusted pairwise comparisons for subcutaneous fat thickness in the lumbar and lower leg regions among postures

Comparison of postures	Mean difference	Standard error	Adjusted *p*-value	95% CI lower limit	95% CI upper limit
Lumbar region					
Sitting vs Side-lying	0.158	0.062	0.072	-0.010	0.327
Sitting vs Prone	0.125	0.062	0.203	-0.043	0.293
Sitting vs Standing	-0.058	0.062	0.781	-0.227	0.110
Side-lying vs Prone	-0.033	0.062	0.948	-0.202	0.135
Side-lying vs Standing	-0.217	0.062	0.008	-0.385	-0.048
Prone vs Standing	-0.183	0.062	0.030	-0.352	-0.014
Lower leg region					
Sitting vs Side-lying	-0.200	0.106	0.429	-0.517	0.117
Sitting vs Supine (knee extension)	-0.175	0.106	0.576	-0.492	0.142
Sitting vs Supine (knee flexion)	0.008	0.106	1.000	-0.308	0.325
Sitting vs Prone	-0.275	0.106	0.123	-0.592	0.042
Sitting vs Standing	-0.041	0.106	0.999	-0.358	0.275
Side-lying vs Supine (knee extension)	0.025	0.106	1.000	-0.292	0.342
Side-lying vs Supine (knee flexion)	0.208	0.106	0.383	-0.108	0.525
Side-lying vs Prone	-0.075	0.106	0.981	-0.392	0.242
Side-lying vs Standing	0.158	0.106	0.675	-0.158	0.475
Supine (knee extension) vs Supine (knee flexion)	0.183	0.106	0.526	-0.133	0.500
Supine (knee extension) vs Prone	-0.100	0.106	0.934	-0.417	0.217
Supine (knee extension) vs Standing	0.133	0.106	0.809	-0.183	0.450
Supine (knee flexion) vs Prone	-0.283	0.106	0.104	-0.600	0.033
Supine (knee flexion) vs Standing	-0.050	0.106	0.997	-0.367	0.267
Prone vs Standing	0.233	0.106	0.263	-0.083	0.550

*p*-values are adjusted for multiple comparisons

## Discussion

The intra-rater reliability for subcutaneous fat thickness measurements in both the lumbar region and the lower leg was very high for both ICC (1, 1) and ICC (1, 3). A significant main effect of posture was observed in the lumbar region, whereas no significant main effect was found in the lower leg after Greenhouse–Geisser correction for violation of sphericity. Post hoc analysis showed that significant pairwise differences were limited to the lumbar region. Across both regions, the observed effect sizes were trivial to small, suggesting that the posture-related differences were limited in magnitude in this sample of healthy young men.

### Reliability

The reliability of the subcutaneous fat thickness measurements in the lumbar and lower leg regions in each posture was very high for both ICC (1, 1) and ICC (1, 3). The reliability of the subcutaneous fat thickness measurements obtained using metal calipers in this study was comparable to that reported in previous studies [10, 11]. The extremely high intra-rater reliability observed in this study is likely due to the consistent application of a standardized skinfold measurement procedure, including consistent identification of the measurement site, caliper placement, timing of reading, and use of the same examiner throughout the study. In addition, cleaning the caliper contact surfaces with alcohol tissues before use was performed as a hygiene and equipment-care procedure, consistent with anthropometric procedure manuals recommending cleaning calipers and related measurement equipment with alcohol or disinfectant wipes [17].

### Effect of posture

ANOVA revealed a significant main effect of posture on subcutaneous fat thickness in the lumbar region. In the lumbar region, post hoc comparisons showed that the standing position yielded slightly greater values than the side-lying and prone positions. The largest lumbar mean difference was 0.217 mm, observed between the side-lying and standing positions. In the lower leg, Mauchly’s test indicated violation of sphericity, and the Greenhouse–Geisser-corrected ANOVA did not show a significant main effect of posture. Therefore, the lower-leg results should not be interpreted as evidence of a clear posture-related difference between specific postures. The largest lower-leg mean difference was 0.283 mm, observed between the supine knee-flexion and prone positions. This difference was small relative to the scale interval of the caliper and may have limited practical relevance; however, it should not be directly interpreted as measurement error or as a minimal detectable change. Measurement error, minimal detectable change, and practical significance are conceptually distinct. Therefore, the present results should be interpreted as indicating small posture-related differences with limited practical magnitude, rather than definitive evidence that the differences fall within measurement error. The effect sizes (Cohen's d) for both the lumbar and lower leg regions (0.03–0.19 and 0.00–0.10, respectively) were very small. In a previous study using ultrasonography, the effect sizes for the lower leg ranged from 0.18 to 0.70 [14]. One possible reason for the discrepancy in effect sizes between the present and previous studies is the difference in participants' BMI; the previous study reported a higher BMI (22.9 ± 2.5) compared with the present study (20.7 ± 4.0). As the participants in the previous study were healthy adult volunteers, a higher BMI likely reflected greater fat accumulation. Because ultrasound imaging is widely used for quantitative assessment of body fat mass [19], differences between ultrasound-based and caliper-based procedures should be considered when interpreting discrepancies across studies. In the previous study, the greatest differences in subcutaneous fat thickness among postures were observed in the lower leg, specifically between the supine position, in which the knees were flexed and no part of the lower limbs was in contact with the surface, and the prone position (supine: 10.9 ± 4.1 mm; prone: 8.2 ± 3.2 mm). Adipose tissue is connected superficially to the dermis and, in deeper layers, to the superficial fascia, the multilayer structure of the deep fascia, and fibrous septa (connective tissue mainly composed of collagen fibers). Muscle fibers are also attached to loose connective tissue and fibrous septa, forming a three-dimensional network of loose connections [20]. Therefore, when the amount of adipose tissue is greater and the skin around the measurement site is in contact with the external surface, or when muscle tension is generated due to loading or posture, tension may develop within the fat layer. This could explain the larger effect sizes reported in the previous study, which involved participants with higher adiposity compared with the present study. In addition to participant characteristics, differences in measurement methods (ultrasound imaging vs. metal calipers) may have contributed to this discrepancy. Measurements obtained with a metal caliper may be influenced by compression of the skinfold and by a potential systematic bias toward overestimation compared with ultrasound imaging [11]. Finally, differences in measurement sites may also have influenced the results (posterior calf gastrocnemius region vs. medial calf region). The previous study described the site as the posterior calf gastrocnemius region without further details; thus, it is possible that site-specific skin tension associated with each posture affected the measurements.

### Limitations

Reproducibility, correlation with ultrasound measurements, and linear regression equations for subcutaneous fat thickness measured using metal calipers have been previously reported [11]. Metal calipers are inexpensive and easy to use compared with ultrasound imaging. However, several limitations should be considered. First, only measurements obtained with a metal caliper were analyzed, and direct comparisons with ultrasound-derived values were not conducted. Therefore, the influence of posture on values closer to the true fat thickness remains unclear. Second, measurement error and minimal detectable change were not directly calculated in the present study. Therefore, small numerical differences should be interpreted in terms of practical magnitude rather than being equated with measurement error. Third, the participants were healthy young men with a relatively low mean BMI, and the influence of posture may differ in individuals with higher adiposity, women, older adults, or clinical populations. It is necessary to examine the effects of posture, particularly in individuals with a BMI ≥ 25 and in those who have difficulty maintaining a standing position. Fourth, all measurements were performed by a single examiner; therefore, inter-rater reliability was not evaluated. Finally, although measurements were performed under air-conditioned room-temperature conditions in a university laboratory, ambient temperature was not recorded for each trial. Future studies should control and report environmental conditions more precisely.

## Conclusions

This study measured subcutaneous fat thickness in the lumbar and lower leg regions using a metal caliper and examined the effects of different postures on measured values and reliability. The reliability of subcutaneous fat thickness measurements was high across all postures in both regions. Small posture-related differences were observed in the lumbar region, whereas no significant main effect was found in the lower leg after correction for violation of sphericity. These findings suggest that alternative body positions may be considered when standing measurement is difficult, provided that the limitations of the present study are recognized. Further studies in women, older adults, individuals with higher BMI, and clinical populations are required before the findings can be generalized broadly.

## Data Availability

The datasets used and/or analysed during the current study are available from the corresponding author on reasonable request.

## Abbreviations

BMI: Body mass index
ICC: Intraclass correlation coefficients
ANOVA: Analysis of variance
CI: Confidence interval

## References

[1] Waongenngarm P, Rajaratnam BS, Janwantanakul P. Internal oblique and transversus abdominis muscle fatigue induced by slumped sitting posture after 1 hour of sitting in office workers. Saf Health Work. 2016;7:49–54. 10.1016/j.shaw.2015.08.001. 27014491 10.1016/j.shaw.2015.08.001 PMC4792914

[2] Srinidhi G, Agrawal KN, Kumari S, Potdar RR, Chandel NS, Rao KV, et al. Muscle fatigue assessment using surface electromyography in farm operations performed in protected cultivation. Sci Rep. 2025;15:33758. 10.1038/s41598-025-00144-w. 41027980 10.1038/s41598-025-00144-w PMC12484682

[3] Felici F, Quaresima V, Fattorini L, Sbriccoli P, Filligoi GC, Ferrari M. Biceps brachii myoelectric and oxygenation changes during static and sinusoidal isometric exercises. J Electromyogr Kinesiol. 2009;19:e1-11. 10.1016/j.jelekin.2007.07.010. 17890107 10.1016/j.jelekin.2007.07.010

[4] Perrey S, Quaresima V, Ferrari M. Muscle oximetry in sports science: an updated systematic review. Sports Med. 2024;54:975–96. 10.1007/s40279-023-01987-x. 38345731 10.1007/s40279-023-01987-x PMC11052892

[5] Kanda M, Kitamura T, Suzuki Y, Konishi I, Watanabe K, Sato N. Intramuscular circulation of the lumbar multifidus in different trunk positions on standing. Adv Exp Med Biol. 2022;1395:405–9. 10.1007/978-3-031-14190-4_66. 36527670 10.1007/978-3-031-14190-4_66

[6] Yasuda A, Kubo K. Effects of static stretching on the blood circulation of human tendon in vivo. Transl Sports Med. 2024;2024:1–8. 10.1155/2024/4413113. 10.1155/2024/4413113 PMC11023726 38654719

[7] Suicmez S, Yazici G, Altiparmak T, Cagatay C, Batur-Caglayan HZ, Nazliel B. The effect of spasticity severity on peripheral muscle oxygenation in hemiparetic stroke patients: a cross-sectional study. Top Stroke Rehabil. 2026;33:164–74. 10.1080/10749357.2025.2538520. 40717605 10.1080/10749357.2025.2538520

[8] Ida A, Sasaki K. Distinct adaptations of muscle endurance but not strength or hypertrophy to low-load resistance training with and without blood flow restriction. Exp Physiol. 2024;109:926–38. 10.1113/EP091310. 38502540 10.1113/EP091310 PMC11140179

[9] Hayes PA, Sowood PJ, Belyavin A, Cohen JB, Smith FW. Sub-cutaneous fat thickness measured by magnetic resonance imaging, ultrasound, and calipers. Med Sci Sports Exerc. 1988;20:303–9. 10.1249/00005768-198806000-00015. 3386511 10.1249/00005768-198806000-00015

[10] Hoffmann J, Thiele J, Kwast S, Borger MA, Schröter T, Falz R, et al. Measurement of subcutaneous fat tissue: reliability and comparison of caliper and ultrasound via systematic body mapping. Sci Rep. 2022;12:15798. 10.1038/s41598-022-19937-4. 36138057 10.1038/s41598-022-19937-4 PMC9500055

[11] Pérez-ChirinosBuxadé C, Solà-Perez T, Castizo-Olier J, Carrasco-Marginet M, Roy A, Marfell-Jones M, et al. Assessing subcutaneous adipose tissue by simple and portable field instruments: skinfolds versus A-mode ultrasound measurements. PLoS ONE. 2018;13:e0205226. 10.1371/journal.pone.0205226. 30496211 10.1371/journal.pone.0205226 PMC6264474

[12] Barata AT, Nunes G, Santos CA, Fonseca J. Clinical use of metal and plastic calipers for nutritional assessment of patients under long-term enteral feeding through endoscopic gastrostomy. Nutr Hosp. 2017;34:1275–80. 10.20960/nh.1069. 29280639 10.20960/nh.1069

[13] International Society for the Advancement of Kinanthropometry. Available from: https://www.isak.global/. Accessed 6 Oct 2025.

[14] Gao Y, Tang X, Li M, Qiu L. Consistency of ultrasound measurements of fat thickness in different postures. Curr Med Imaging. 2025;21:e15734056369864. 10.2174/0115734056369864250725081342. 40754883 10.2174/0115734056369864250725081342 PMC13223413

[15] Marfell-Jones M, Olds T, Stewart A, Carter L. International standards for anthropometric assessment. Potchefstroom: International Society for the Advancement of Kinanthropometry; 2006.

[16] Surface ElectroMyoGraphy for the Non-Invasive Assessment of Muscles (SENIAM). Available from: https://seniam.org/. Accessed 10 Oct 2025.

[17] Centers for Disease Control and Prevention. National health and nutrition examination survey: anthropometry procedures manual. Hyattsville, MD: National Center for Health Statistics; 2007.

[18] Krämer HJ, Ulmer HV. Two-second standardization of the Harpenden caliper. Eur J Appl Physiol Occup Physiol. 1981;46:103–4. 10.1007/BF00422182. 7194782 10.1007/BF00422182

[19] Gao Y, Tang X, Liu B, Qiu L. Application of ultrasound for quantitative assessment of body fat mass. Clin Nutr ESPEN. 2025;67:635–44. 10.1016/j.clnesp.2025.03.175. 40204071 10.1016/j.clnesp.2025.03.175

[20] Stecco C, Tiengo C, Stecco A, Porzionato A, Macchi V, Stern R, et al. Fascia redefined: anatomical features and technical relevance in fascial flap surgery. Surg Radiol Anat. 2013;35:369–76. 10.1007/s00276-012-1058-0. 23266871 10.1007/s00276-012-1058-0

